# The Role of Apolipoprotein E as a Risk Factor for an Earlier Age at Onset for Machado-Joseph Disease Is Doubtful

**DOI:** 10.1371/journal.pone.0111356

**Published:** 2014-11-04

**Authors:** Qi Zhou, Wang Ni, Yi Dong, Ning Wang, Shi-Rui Gan, Zhi-Ying Wu

**Affiliations:** 1 Department of Neurology and Institute of Neurology, Second Affiliated Hospital, School of Medicine, Zhejiang University, Hangzhou, Zhejiang, China; 2 Department of Neurology and Institute of Neurology, First Affiliated Hospital, Fujian Medical University, Fuzhou, Fujian, China; 3 Department of Neurology and Institute of Neurology, Huashan Hospital, Shanghai Medical College, Fudan University, Shanghai, China; Emory University, United States of America

## Abstract

Machado-Joseph disease (MJD) is an inherited neurodegenerative disease caused by an expanded CAG repeat in the *ATXN3* gene. Although the principal genetic determinant of the age at onset (AAO) is the length of the expanded CAG repeat, the additional genetic contribution of MJD toward the AAO has mostly not yet been clarified. It was recently suggested in two independent studies that apolipoprotein E (*APOE*) might be associated with AAO variability in MJD patients. To identify the potential modifier effect of *APOE* polymorphisms on the AAO of MJD patients, 403 patients with MJD (confirmed by molecular tests) from eastern and southeastern China were enrolled in the present study. CAG repeats in the *ATXN3* and *APOE* polymorphisms were genotyped. Data were analyzed using a statistical package. No contribution of *APOE* polymorphisms to the variance in disease onset was observed using ANCOVA (F = 0.183, P = 0.947). However, significant effects on the AAO of MJD were found for the normal *ATXN3* allele and for the interaction of mutant and normal *ATXN3* alleles in a multiple linear regression model (P = 0.043 and P = 0.035, respectively). Our study does not support a role for *APOE* as a genetic modifier of the AAO of MJD. Additionally, our study presents evidence that the normal *ATXN3* allele and its interaction with mutant alleles contribute toward AAO variance in MJD patients.

## Introduction

Machado-Joseph disease (MJD) is an autosomal dominant neurodegenerative disorder characterized by cerebellar ataxia associated with marked phenotypic heterogeneity [1]. The disease usually starts during adulthood, but the age at onset (AAO) ranges from adolescence to old age [2]. MJD is caused by a CAG repeat expansion in exon 10 of the *ATXN3* gene that translates to an elongated polyglutamine tract in the ataxin-3 protein [3]. Similar to several other polyglutamine disorders, the AAO for MJD patients is not completely determined by the expanded CAG repeat. Previous studies have demonstrated that the length of the mutant CAG repeat explains approximately 45–87% of the total AAO variance of MJD [4]–[6]. Familial factors are responsible for some of the residual variance [5], [7], indicating that modifier genes may play a role in the AAO variance. The identification and characterization of these modifiers offer an important opportunity to better understand the biological mechanisms involved in the disease, improving consultation services for pre-symptomatic individuals.

The human apolipoprotein E (*APOE*) gene is polymorphic, with three major alleles (ε2, ε3, and ε4) that differ from each other at two crucial non-synonymous sites [8], [9]. Therefore, the corresponding three isoforms, E2, E3, and E4, ultimately possess distinct structural and functional properties [10]. The *APOE* polymorphisms have been investigated in a number of neurodegenerative diseases with respect to risk, progression, and AAO [11]–[14]. In particular, the association of *APOE* with the AAO for Huntington's disease (HD) was reported in several studies, but the results were inconsistent [15]–[19].

Recently, two independent groups reported an association of the *APOE* ε2 allele with an earlier AAO for MJD in small samples (fewer than 200 patients) [20], [21]. To further investigate this issue, we collected a large cohort of MJD patients (more than 400 individuals) from eastern and southeastern China and then analyzed the association of *APOE* polymorphisms with the AAO.

## Materials and Methods

### MJD patients

In the present study, 403 patients from 362 unrelated families with molecular confirmation of MJD were enrolled. The patients were surveyed between February 2005 and March 2013, and they were all from eastern and southeastern China. Each patient gave informed consent, and the local ethics committees approved the study protocol. AAO was defined as the age at which the first symptoms of ataxia occurred. Whenever possible, the AAO was corroborated by close relatives or care providers.

### Molecular analyses

Genomic DNA was extracted from peripheral blood using the QIAamp DNA Blood Mini Kit (Qiagen, Hilden, Germany) following standard procedures. Molecular analysis of *ATXN3* was performed as previously reported [22], and the lengths of the expanded and normal CAG repeats were determined using Sanger sequencing. The *APOE* polymorphisms (SNP combinations of rs429358 and rs7412) were determined using fluorescence-based polymerase chain reaction-restriction fragment length polymorphism analysis. Primer pairs were designed to genotype rs429358 (fluorescence-labeled forward primer: 5′-[FAM] AGG GCG CTG ATG GAC GAG AC-3′ and reverse primer 5′- GCC CCG GCC TGG TAC ACT-3′) and rs7412 (fluorescence-labeled forward primer 5′-[FAM] GGC GCG GAC ATG GAG GAC-3′ and reverse primer 5′-GCC CCG GCC TGG TAC ACT-3′). PCR reactions for the two SNPs were each performed in a 10 µL reaction mixture containing 1x GC buffer I, 2 mM MgCl2, 0.2 mM dNTP, 2 µM primers, 1 U Hotstar Taq polymerase (Qiagen, Hilden, Germany), and 30 ng template DNA, using the following cycling conditions: an initial denaturation at 95°C for 15 min, followed by 11 cycles of 94°C for 20 s, 65°C (0.5°C decrease per cycle) for 40 s, 72°C for 1.5 min, then another 24 cycles of 94°C for 20 s, 59°C for 30 s, and 72°C for 1.5 min, and a final extension step at 72°C for 2 min. The amplification products were then incubated with the endonucleases AflIII (1 U) and HaeII (1 U) (New England Biolabs, Beverly, MA, USA), respectively, according to the manufacturer's recommendations. Finally, the corresponding digestion products and a pair of control fragments were collectively detected using automated capillary electrophoresis in an ABI PRISM 3130 Genetic Analyzer (Applied Biosystems).

### Statistical analyses

All statistical analyses were performed using commercially available software (SPSS, version 17.0, downloaded for free from http://www.stathome.cn/html/down/ruanjian/2009/0601/435.html). A p value <0.05 (two-tailed) was considered to be statistically significant. The variability in the AAO attributable to the expanded CAG repeat was calculated using linear regression. Either Fisher's exact test or a chi-square test was used to compare the distribution frequency of *APOE* genotypes or alleles between our cohort and the cohorts reported by Bettencourt et al. [20] and Peng et al. [21]. Differences in the AAO according to the *APOE* genotype and gender of the patient were examined using a two-tailed t-test or an ANCOVA, adjusted for the expanded *ATXN3* allele. Multiple linear regressions were used to calculate the effect of several factors on the AAO: the number of CAG repeats in the expanded and normal *ATXN3* alleles, their interaction, the *APOE* status, and the patient gender. To further validate the association between *APOE* polymorphisms and the AAO in our sample of 403 MJD patients, they were sorted according to gender. Differences in the AAO according to the *APOE* genotype of each group were examined using ANCOVA after adjusting for the expanded *ATXN3* allele. Furthermore, to reduce potential deviation encountered while defining the AAO, differences in the AAO according to the *APOE* genotype in 225 MJD patients with a shorter duration of disease (less than or equal to 5 years) were examined using ANCOVA after adjusting for the expanded *ATXN3* allele.

## Results

The average AAO (±SE) of this cohort of 403 MJD patients (211 males and 192 females) was 36.28 (±0.56) years (range: 10–72 years). The average CAG repeat size (±SE) was 75.90 (±0.186) repeats (range: 53–87 repeats). A chromatogram of an MJD patient with 14/86 CAG repeats is shown in **Figure S1**. The capillary electrophoresis analysis of *APOE* polymorphisms is shown in **Figure S2**.

As expected, the number of expanded CAG repeats was negatively associated with the AAO (Pearson R^2^ = 0.659, p = 0.000, **Figure S3**), in accordance with previous studies [4]–[6]. The characteristics of this patient cohort are presented in **Table 1**. There was no significant difference in the genotypic or allelic distributions of *APOE* between the present cohort and the cohorts reported by Bettencourt et al. [20] (genotype: p = 0.789; allele: p = 0.501) and Peng et al. [21] (genotype: p = 0.44; allele: p = 0.872). Patients carrying different *APOE* genotypes showed no differences in the AAO adjusted for the expanded *ATXN3* allele (ANCOVA, F = 0.18, p = 0.9474). Furthermore, the gender of the patients did not affect the AAO according to either a two-tailed t-test (p = 0.087) or an ANCOVA when using the number of expanded CAG repeats as a covariate (F = 0.435, p = 0.510).

**Table 1:** Characteristics of the studied series of MJD patients.

Variables	*APOE* Genotype					Total
	ε2/ε3	ε3/ε3	ε3/ε4	ε2/ε4	ε4/ε4	
Number of patients (%)	53 (13.2)	263 (65.3)	75 (18.6)	9 (2.2)	3 (0.7)	403 (100)
Gender, No. (%)						
Male	33 (15.6)	130 (61.6)	40 (19.0)	6 (2.8)	2 (0.9)	211 (100)
Female	20 (10.4)	133 (69.3)	35 (18.2)	3 (1.6)	1 (0.5)	192 (100)
Age at onset, y						
Mean±SE [range]	34.14 (1.40)	36.12 (0.67)	38.32 (1.56)	35.89 (2.53)	39.17 (1.88)	36.28 (0.56)
	[10–54]	[14–59]	[13–72]	[24–46]	[36–42]	[10–72]
Adjusted onset^a^ ± SE	35.90 (0.91)	36.29 (0.41)	36.51 (0.76)	35.59 (2.20)	38.59 (3.81)	
CAG repeat length, No.						
Normal, mean (SE) [range]	18.92 (0.92)	19.86 (0.43)	19.59 (0.82)	22.89 (2.60)	14.00 (0.00)	19.71 (0.346)
	[14–34]	[14–38]	[14–37]	[14–35]	14	[14–38]
Expanded, mean (SE) [range]	76.62 (0.48)	75.97 (0.21)	75.16 (0.57)	75.78 (0.46)	75.67 (0.88)	75.90 (0.186)
	[67–87]	[60–84]	[53–86]	[74–79]	[74–77]	[53–87]

a Adjusted for the average expanded CAG repeat length in the studied series of MJD patients.

Compared to the linear model that included only the number of CAG repeats in the abnormal allele, multiple linear regression analysis increased the predictive value of the age at onset by an additional 0.7% (from 65.9% to 66.6% of variance in the AAO). From this model, we found a significant contribution to the variance in the AAO from the number of CAG repeats in the expanded (p = 0.000) and normal alleles (p = 0.043), as well as from their interaction (p = 0.035). Both the *APOE* genotype and the patient gender failed to show significant effects on the AAO in this model (p = 0.892 and p = 0.512, respectively), in agreement with the results of the ANCOVA.

When we sorted the MJD patients according to gender to validate the association between *APOE* polymorphisms and the AAO, the results indicated that there was no sex specific influence of *APOE* genotype (ANCOVA, male: p = 0.915; female: p = 0.849); Furthermore, our evaluation of the 225 MJD patients with a shorter duration of disease indicated that there were no differences in the AAO according to the *APOE* genotype (ANCOVA, p = 0.627).

## Discussion

The underlying pathophysiology of MJD is complex and remains unclear. Association studies of candidate genes assumed to be involved in MJD pathogenesis provide a good opportunity to obtain useful clues for understanding MJD pathogenesis. It is well established that *APOE* plays an important role in mediating cholesterol metabolism and regulating neural growth, regeneration, and repair in the central nervous system. We were therefore motivated to investigate the effect of *APOE* polymorphisms on the AAO in a large cohort of Chinese Han MJD patients. Unfortunately, our study failed to show a significant association between the *APOE* ε2 allele and the AAO for MJD, although we analyzed a cohort of patients larger than those of previous studies [20], [21]. To minimize the influences of gender and disease duration on the results, all patients were further stratified by sex and disease duration and then re-examined; however, the results were still negative. There are several possible explanations for this discrepancy.

First, the sizes of the studied samples vary. The sample size of the present study is twice as large as the sample sizes of the previous studies [20], [21]. Similarly, among association studies of *APOE* that indicated an earlier AAO for HD, the sample sizes were larger in the negative reports [17]–[19] than in the positive reports [15], [16]. It should be noted that only prospective studies are capable of obtaining precise data with respect to the AAO. Despite this limitation, we can achieve a decent approximation using a stringent definition of the AAO and a larger sample size. In both of the previous studies [20], [21], no more than 20 MJD patients carried the ε2/ε3 genotype, which may have led to a somewhat biased estimation of the mean AAO in MJD patients. However, 53 patients carried the ε2/ε3 genotype in our cohort, approximately three times as many compared with previous studies [20], [21].

Second, the genetic heterogeneity across populations could account for the discrepancy, including differences in genotypic distributions and the underlying linkage disequilibrium (LD) patterns. Compared with the findings of Bettencourt et al. [20] and Peng et al. [21], we did not observe a significant difference in the genotypic distributions of *APOE*. Therefore, the lack of association with the *APOE* ε2 allele may be due to different LD structures in the region surrounding *APOE* across populations. In other words, there may be other functional polymorphisms that lead to early AAO in strong LD with the *APOE* ε2/ε3 genotype in the patients examined by Bettencourt et al. [20] and Peng et al. [21], whereas a similar LD pattern simply does not exist in our cohort from the Han population of eastern and southeastern China.

Third, because genetic interaction with various environments may produce distinct phenotypes, the standard of living and other environmental factors may also modulate the effects of *APOE* polymorphisms on the AAO of MJD patients. Additionally, the present results of the multiple regression analysis suggested that the normal *ATXN3* allele and the interaction of the mutant and normal *ATXN3* alleles contribute to the AAO variance in MJD. Similarly, several studies reported the same results in MJD [6], [23] and HD [24], [25]. It was observed that the processes of aggregation, nucleation, and cytotoxicity of mutational polyglutamine proteins were exacerbated by their normal counterparts in a *Drosophila* model [26]. Specific analyses of the underlying mechanism of this interaction in MJD requires further elucidation.

In conclusion, our study casts doubt on the role of *APOE* as a risk factor for an earlier age at onset for MJD. The normal *ATXN3* allele and its interaction with mutant alleles contribute toward AAO variance in MJD patients.

## Supporting Information

Figure S1 **Chromatogram of MJD patients with CAG repeats of 14/86.** Normal CAG repeat expansion is indicated by a thick black arrow, whereas the abnormal CAG repeat sequence is colored in gray.
(JPG)

Figure S2 Capillary electrophoresis analysis of the genotypes of APOE*. *The *APOE* polymorphisms are the SNP combinations rs429358 and rs7412. The amplification products for rs429358 that could be cleaved by the AflIII endonuclease into fluorescently labeled 153-bp and non-fluorescently labeled 164-bp fragments indicated allele T (capillary electrophoresis analysis revealed one peak at 153 bp), whereas the products that could not be cleaved showed a 317-bp peak in the capillary electrophoresis analysis and indicated allele C. Similarly, the amplification products for rs7914 that could be cleaved by the HaeII endonuclease into fluorescently labeled 162-bp and non-fluorescently labeled 23-bp fragments indicated allele C (capillary electrophoresis analysis revealed one peak at 162 bp), whereas those that could not be cleaved showed a 185-bp peak in the capillary electrophoresis analysis and indicated allele T.
(JPG)

Figure S3 **Negative correlation between the age at onset and the number of expanded CAG repeats in MJD (n = 403, R^2^ = 0.659).** The regression line is Y = -2.4X+222 (Y: age of onset, X: number of expanded CAG repeats).
(JPG)
