# Supplementary figures and images for: The Role of Apolipoprotein E as a Risk Factor for an Earlier Age at Onset for Machado-Joseph Disease Is Doubtful
Source: PLoS One. 2014 Nov 4;9(11):e111356. doi: 10.1371/journal.pone.0111356 (PMC4219713; doi:10.1371/journal.pone.0111356)

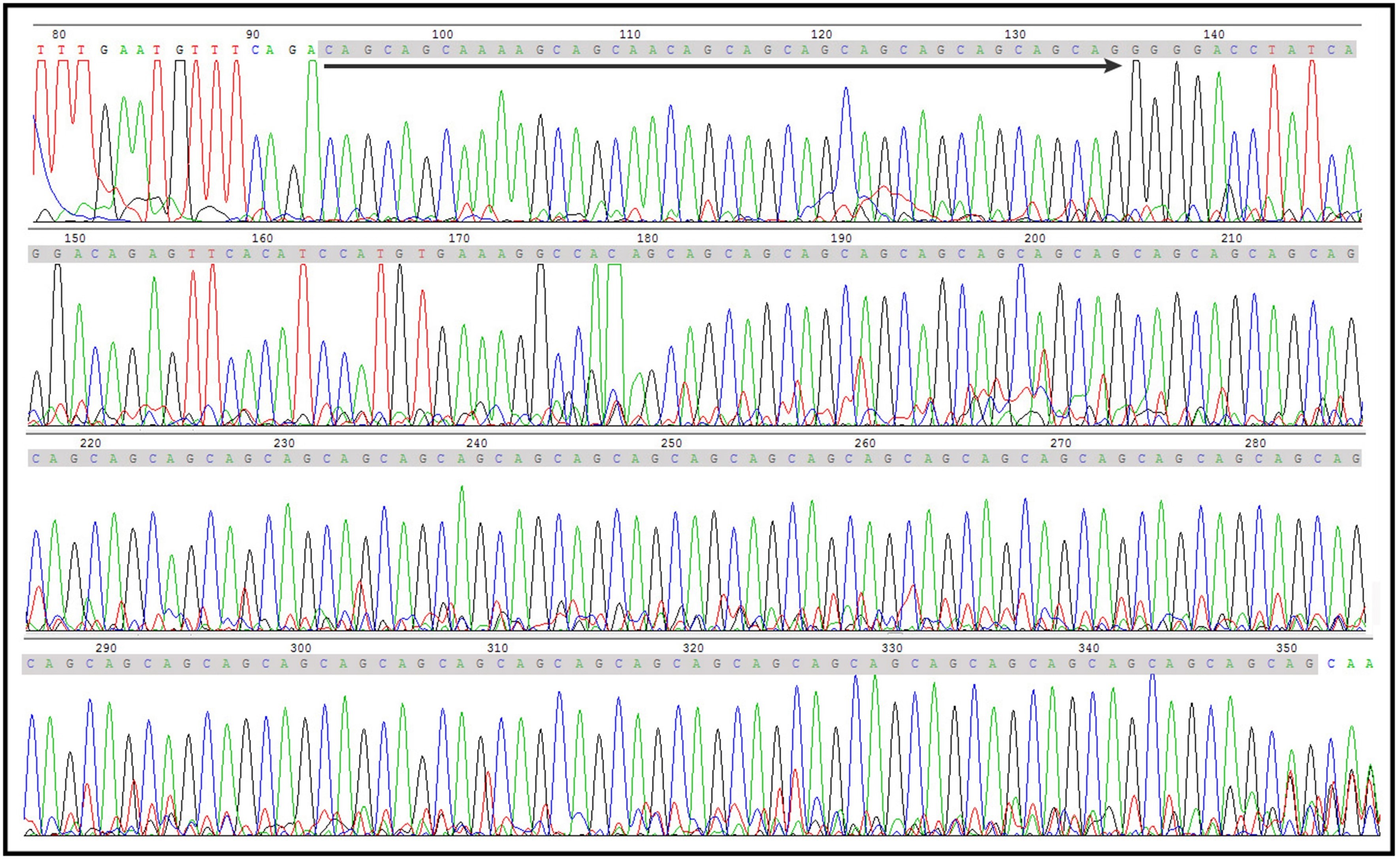

Supplement: Figure S1 — Chromatogram of MJD patients with CAG repeats of 14/86. Normal CAG repeat expansion is indicated by a thick black arrow, whereas the abnormal CAG repeat sequence is colored in gray. (JPG) [file pone.0111356.s001.jpg]

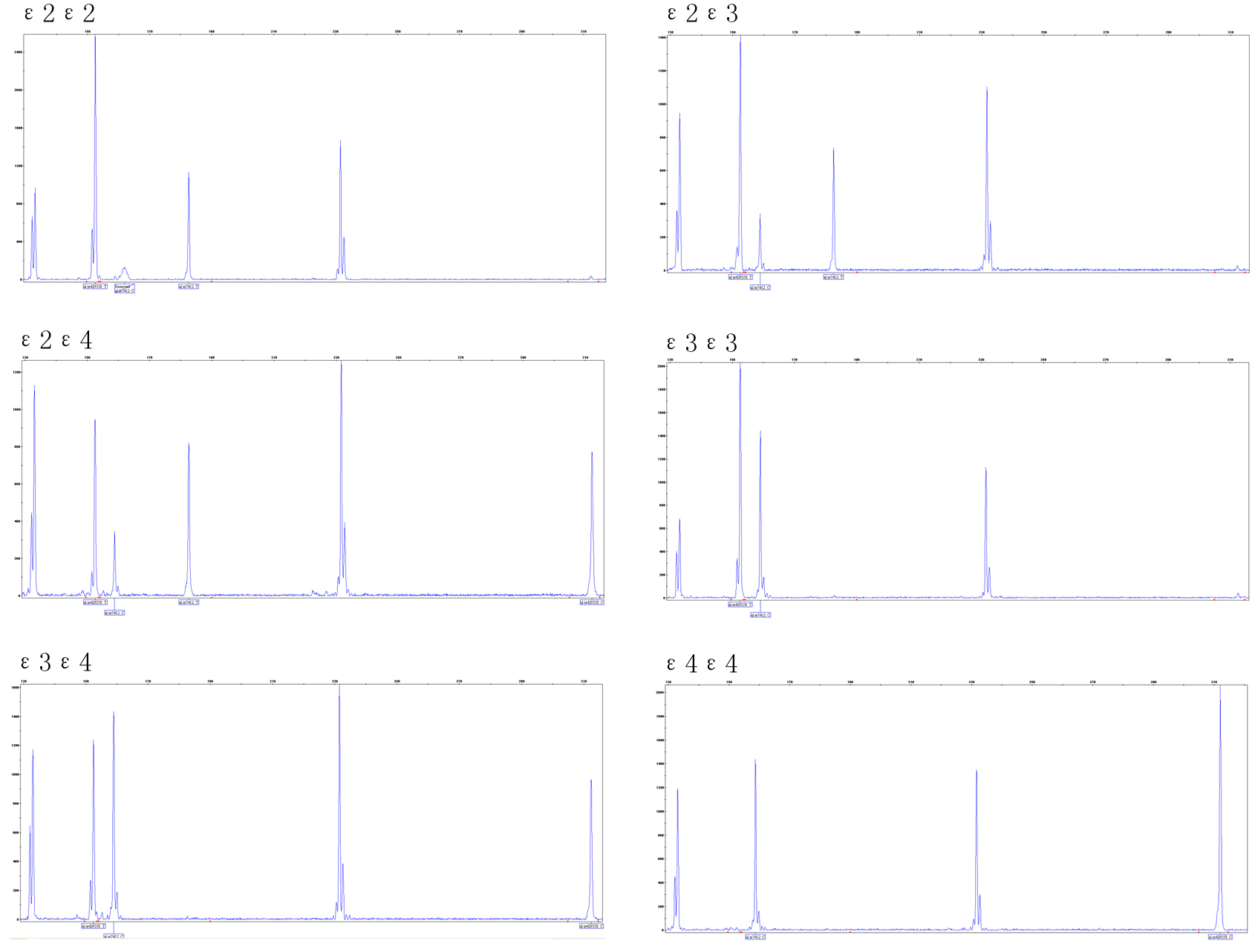

Supplement: Figure S2 — Capillary electrophoresis analysis of the genotypes of APOE*. *The APOE polymorphisms are the SNP combinations rs429358 and rs7412. The amplification products for rs429358 that could be cleaved by the AflIII endonuclease into fluorescently labeled 153-bp and non-fluorescently labeled 164-bp fragments indicated allele T (capillary electrophoresis analysis revealed one peak at 153 bp), whereas the products that could not be cleaved showed a 317-bp peak in the capillary electrophoresis analysis and indicated allele C. Similarly, the amplification products for rs7914 that could be cleaved by the HaeII endonuclease into fluorescently labeled 162-bp and non-fluorescently labeled 23-bp fragments indicated allele C (capillary electrophoresis analysis revealed one peak at 162 bp), whereas those that could not be cleaved showed a 185-bp peak in the capillary electrophoresis analysis and indicated allele T. (JPG) [file pone.0111356.s002.jpg]

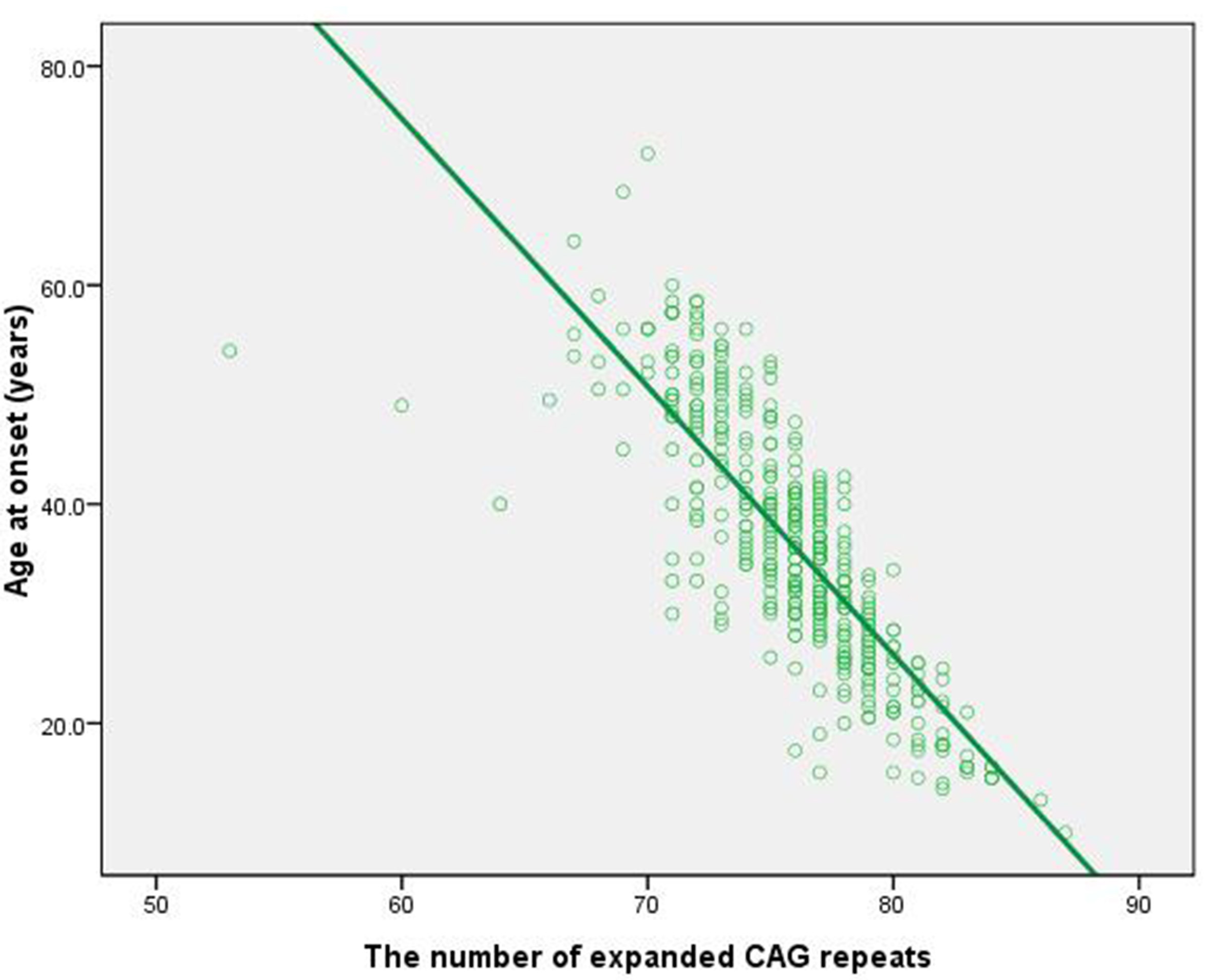

Supplement: Figure S3 — Negative correlation between the age at onset and the number of expanded CAG repeats in MJD (n = 403, R2 = 0.659). The regression line is Y = -2.4X+222 (Y: age of onset, X: number of expanded CAG repeats). (JPG) [file pone.0111356.s003.jpg]
